# Daumone fed late in life improves survival and reduces hepatic inflammation and fibrosis in mice

**DOI:** 10.1111/acel.12224

**Published:** 2014-05-06

**Authors:** Jong Hee Park, Hae Young Chung, Minkyu Kim, Jung Hwa Lee, Mankil Jung, Hunjoo Ha

**Affiliations:** 1 Graduate School of Pharmaceutical Sciences, College of Pharmacy, Ewha Womans University, Global Top 5 Research Program52 Ewhayoedae-gil, Seodaemun-gu, Seoul, Korea; 2 Aging Tissue Bank, Department of Pharmacy, College of Pharmacy, Pusan National University2 Busandaehak-ro 63beon-gil, Geumjeong-gu, Busan, Korea; 3 Department of Chemistry, Yonsei University50 Yonsei-ro, Seodaemun-gu, Seoul, Korea

**Keywords:** aging, inflammation, insulin resistance, liver, longevity, pheromones

## Abstract

The liver is one of the most susceptible organs to aging, and hepatic inflammation and fibrosis increase with age. Chronic inflammation has been proposed as the major molecular mechanism underlying aging and age-related diseases, whereas calorie restriction has been shown to be the most effective in extending mammalian lifespan and to have anti-aging effects through its anti-inflammatory action. Thus, it is necessary to develop effective calorie restriction mimetics. Daumone [(2)-(6R)-(3,5-dihydroxy-6-methyltetrahydropyran-2-yloxy)heptanoic acid], a pheromone secreted by *Caenorhabditis elegans*, forces them to enter the dauer stage when facing inadequate conditions. Because *Caenorhabditis elegans* live longer during the dauer stage under energy deprivation, it was hypothesized that daumone may improve survival in mammals by mimicking calorie restriction. Daumone (2 mg kg^−1^ day^−1^) was administered orally for 5 months to 24-month-old male C57BL/6J mice. Daumone was found to reduce the risk of death by 48% compared with age-matched control mice, and the increased plasma insulin normally presented in old mice was significantly reduced by daumone. The increased hepatic hypertrophy, senescence-associated β-galactosidase activity, insulin resistance, lipid accumulation, inflammation, oxidative stress, and fibrosis in old mice were significantly attenuated by daumone. From a mechanistic view, daumone reduced the phosphorylation of the IκBα and upregulation of *Rela* and *Nfkbia* mRNA in the livers of old mice. The anti-inflammatory effect of daumone was confirmed in lipopolysaccharide-induced liver injury model. Oral administration of daumone improves survival in mice and delivers anti-aging effects to the aged liver by modulating chronic inflammation, indicating that daumone could be developed as an anti-aging compound.

## Introduction

Aging is defined as any change in an organism that occurs over time and results in the accumulation of physiological and pathological changes. Importantly, it has been shown that homeostasis is not maintained during the process of aging, and the liver is one of the most susceptible organs to aging (Le Couteur *et al*., 2010). Age-related morphological and functional changes that occur in human and experimental animal livers include reduced hepatic blood flow, accumulated lipids, swollen stellate cells that store fat and produce extracellular matrix (ECM), decreased antioxidants, and increased inflammation (Le Couteur *et al*., 2010). Alanine transaminase (ALT) and aspartate transaminase (AST) are also increased in aged mice (Gregg *et al*., 2012).

Diverse theories have been proposed to describe the underlying mechanisms of aging (Lopez-Otin *et al*., 2013). However, these theories are not mutually exclusive and may be related. Inflammation has been proposed as an important molecular mechanism of age-related liver disease and aging (Le Couteur *et al*., 2010). Continuous overstimulation of the immune system, a defense against external stress, over time results in chronic inflammation, which leads to aging (Chung *et al*., 2011). In fact, healthy centenarians have lower serum levels of inflammatory cytokines (Ferrucci *et al*., 1999), and proinflammatory molecules such as interleukin-1β (IL-1β), IL-6, and tumor necrosis factor-α (TNF-α) are upregulated in the aged liver (Rikans *et al*., 1999). However, SRT1720, an anti-aging SIRT1 activator, suppresses proinflammatory gene expressions in the livers of aged mice fed a standard diet (Mitchell *et al*., 2014) as well as a high-fat diet (Minor *et al*., 2011). Although the precise mechanism of age-related inflammation remains elusive, it is important to inhibit inflammation to prevent liver aging.

Calorie restriction (CR) and anti-aging agents have been reported to increase the lifespan of diverse organisms, including yeast, worms, mice, and monkeys, although CR has not yet been demonstrated to extend the human lifespan (Fontana *et al*., 2010). CR and other anti-aging agents protect against age-related diseases, such as cardiovascular diseases in humans (Fontana *et al*., 2010). Furthermore, CR reduces the serum ALT levels in patients with chronic liver disease (Iwasa *et al*., 2010) and serum proinflammatory cytokines in mice (Rahman *et al*., 2009). Thus, it would be beneficial to identify effective CR mimetics. Resveratrol (Baur *et al*., 2006) and SRT1720 (Mitchell *et al*., 2014), SIRT1 activators, and rapamycin (Harrison *et al*., 2009), a mammalian target of rapamycin (mTOR) inhibitor, have been proposed as candidate CR mimetics. Resveratrol improves survival and decreases age-related changes such as insulin resistance, mitochondrial number, the motor dysfunction, and liver pathology in mice fed a high-calorie diet (Baur *et al*., 2006). Resveratrol also reduced hepatic steatosis, the levels of reactive oxygen species, and inflammation in high-fat-diet-induced obese mice (Jeon *et al*., 2012). SRT1720 extends lifespan, delays onset of age-related metabolic disease, and improves general health in male mice (Mitchell *et al*., 2014). Rapamycin was shown to extend the lifespan of mice fed a standard diet (Harrison *et al*., 2009; Miller *et al*., 2011), but it has been reported to induce hyperlipidemia and glucose intolerance (Houde *et al*., 2010). A very recent study found that metformin, an adenosine monophosphate-activated protein kinase (AMPK) activator, improved the health span in male mice (Martin-Montalvo *et al*., 2013).

The dauer pheromone is secreted by *Caenorhabditis elegans (C. elegans)* under the conditions of starvation, inadequate temperature, or overcrowding (Hu, 2007). The dauer pheromone was initially identified as a fatty acid-like pheromone and was partially purified (Golden & Riddle, 1982). In 2005, we successfully synthesized the dauer pheromone, daumone [(2)-(6R)-(3,5-dihydroxy-6-methyltetrahydropyran-2-yloxy)heptanoic acid], and confirmed its ability to induce dauer formation (Jeong *et al*., 2005). When *C. elegans* enter the dauer stage, their lifespan is extended, and the changes observed in the gene expression profiles of *C. elegans* in response to daumone were consistent with the induction of a mammalian anti-aging program, including decreased mTOR signaling, decreased transforming growth factor-β1 (TGF-β1), increased mitochondrial defense mechanisms, and attenuated oxidative stress (Jeong *et al*., 2009). Because dauer larva formation is stimulated by starvation, we hypothesized that daumone may act as a CR mimetic in mammals.

In this study, we demonstrated that daumone improves survival in mice. We also identified anti-aging effects of daumone, including reduced hepatic senescence, inflammation, fibrosis, and lipid accumulation, and improved hepatic insulin signaling and systemic insulin sensitivity. In addition, daumone had protective effect on lipopolysaccharide (LPS)-induced liver injury in young mice, supporting its anti-inflammatory effect.

## Results

### Reduced risk of death in daumone-treated aged mice

Old mice received daumone for 5 months from the age of 24 months by oral gavage. Twenty-nine-month-old mice demonstrated significantly increased liver weights and plasma ALT compared with 10-week-old mice. The levels of plasma insulin, cholesterols, triglycerides, free fatty acids, and lipid peroxides (LPO) were also increased in old mice compared with young mice. Daumone effectively lessened aging-induced liver hypertrophy and plasma ALT, insulin, triglycerides, and LPO (Table 1). Interestingly, high-density lipoprotein (HDL) cholesterol was increased by daumone.

**Table 1:** General characteristics of experimental animals

	Y	O	O+D
Body weight (g)	25.3 ± 0.3	33.4 ± 1.6*	33.1 ± 0.7*
Liver weight (g)	1.2 ± 0.0	2.9 ± 0.4*	1.8 ± 0.1*†
Liver weight/body weight (g kg^−1^)	46.4 ± 1.0	82.1 ± 11.4*	54.8 ± 2.8*†
Plasma ALT (IU L^−1^)	18 ± 2	60 ± 14*	38 ± 4*†
Plasma insulin (ng mL^−1^)	0.56 ± 0.08	0.95 ± 0.02*	0.63 ± 0.06†
HbA1c (%)	4.3 ± 0.1	4.2 ± 0.1	4.1 ± 0.1
Plasma triglyceride (μm)	140 ± 11	179 ± 19*	136 ± 14†
Plasma total cholesterol (mg dL^−1^)	33 ± 5	69 ± 4*	76 ± 6*
Plasma HDL cholesterol (mg dL^−1^)	19 ± 1	40 ± 1*	57 ± 5*†
Plasma LDL cholesterol (mg dL^−1^)	10 ± 1	36 ± 7*	32 ± 2*
Plasma glucose (mg dL^−1^)	208 ± 7	130 ± 23*	129 ± 12*
Plasma free fatty acid (μm)	29 ± 5	1162 ± 441*	1013 ± 212*
Plasma lipid peroxides (μm)	4.3 ± 0.5	13.2 ± 2.6*	7.6 ± 1.3*†

Data are presented as the mean±SE of 7–17 mice/group. ALT, alanine transaminase; HDL, high-density lipoprotein; LDL, low-density lipoprotein; Y, young mice; O, old mice; O+D, old mice treated with daumone (2 mg kg^−1^).

* *P* < 0.05 vs. Y.

† *P* < 0.05 vs. O.

**Figure 1:**
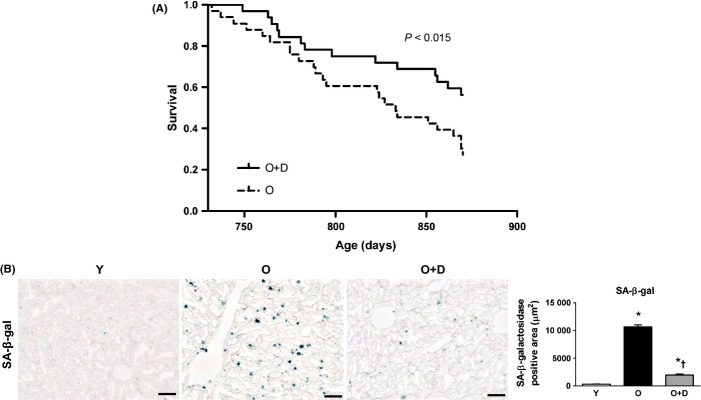
Effects of daumone on survival and hepatic senescence in aged mice (A) A Kaplan–Meier survival curve. Daumone or vehicle (drinking water) was administered to 32 mice in each group. (B) Senescent cells were detected by senescence-associated β-galactosidase (SA β-gal) staining of liver tissues, and the positive area was quantitated using ImageJ. Data are presented as the mean±SE of 4 mice/group. *P < 0.05 vs. Y, †*P* < 0.05 vs. O. Y, young mice; O, old mice; O+D, old mice treated with daumone (2 mg kg^−1^). Magnification, 200×; scale bar, 50 μm.

A Kaplan–Meier survival analysis demonstrated that daumone improved survival in mice (Fig. 1A). A Cox proportional hazards regression demonstrated that daumone reduced the risk of death by 48%.

### Decreased hepatic-senescence-associated β-galactosidase (SA-β-gal) activity in daumone-treated aged mice

Hepatic SA-β-gal activity, a marker of senescence, was barely detectable in young mice and was significantly increased in old mice (Fig. 1B). Daumone effectively reduced the SA-β-gal activity in the livers of old mice, suggesting that age-related hepatic senescence could be attenuated by daumone.

### Attenuated liver inflammation in daumone-treated aged mice

**Figure 2:**
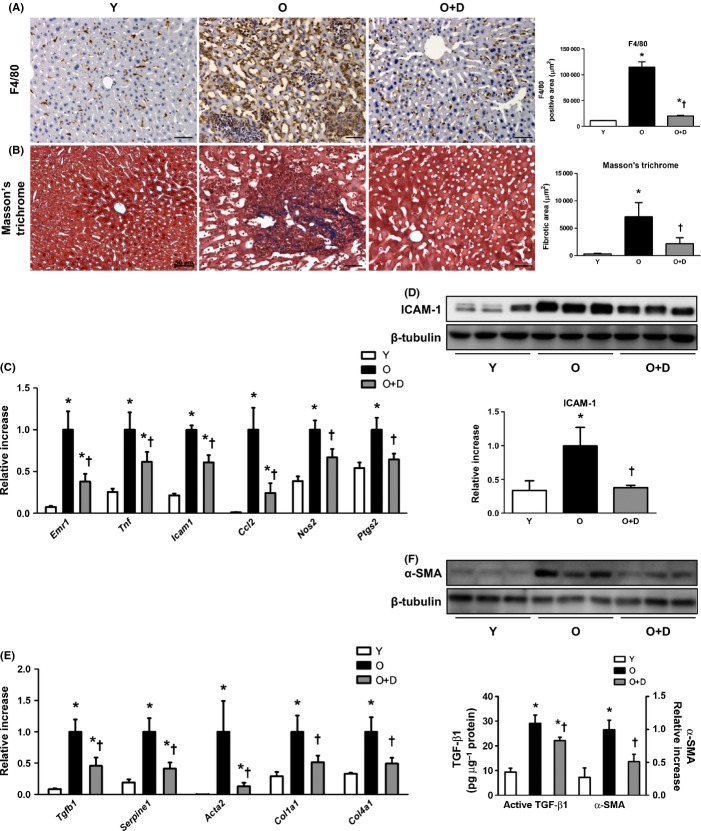
Effects of daumone on hepatic inflammation and fibrosis in aged mice. (A) Macrophage infiltration was detected by F4/80 immunohistochemical staining. Brown, F4/80; blue, hematoxylin. (B) Collagen was stained with Masson’s trichrome staining. The positive area was quantitated using Image-Pro Plus and presented as the mean±SE of 4 mice/group. (C, E) mRNA levels were determined by real-time qRT–PCR. (D, F) Immunoblots of the liver are presented and quantitated. Transforming growth factor-β1 (TGF-β1) was measured by ELISA. mRNA and protein levels were presented as the mean±SE of 7–17 mice/group. *P < 0.05 vs. Y, †*P* < 0.05 vs. O. Y, young mice; O, old mice; O+D, old mice treated with daumone. Magnification, 200×; scale bar, 50 μm.

Macrophage infiltration, as measured by F4/80 staining, was markedly increased in old mice compared with young mice; however, this increased infiltration was effectively reduced by daumone (Fig. 2A). Moreover, the mRNA levels of *Emr1* (F4/80) were significantly increased in old mice, and this increase was reduced by daumone (Fig. 2C).

Similarly, the mRNA levels of inflammatory cytokines, namely *Tnf*, *Icam1*, *Ccl2*, *Nos2*, and *Ptgs2*, were significantly increased in aged mouse livers as compared to young livers, and this increase was significantly reduced by daumone (Fig. 2C). The protein levels of intercellular adhesion molecule-1 (ICAM-1) were significantly increased in old mice compared with young mice (Fig. 2D), and this increase was inhibited by daumone.

### Attenuated fibrosis in daumone-treated aged mice

ECM accumulation and fibrosis are well-established markers of the aging liver (Le Couteur *et al*., 2010). To confirm ECM accumulation, Masson’s trichrome staining was performed. The amount of collagen in the livers of aged mice was increased compared with that of young mice, and this increase was significantly reduced by daumone (Fig. 2B). The mRNA levels of ECM components, such as *Col1a1* and *Col4a1*, and fibrosis markers, such as *Tgfb1*, *Serpine1*, and *Acta2*, were significantly increased in aged mouse livers (Fig. 2E). The protein levels of TGF-β1 and α-smooth muscle actin (α-SMA) were also significantly increased in aged mouse livers (Fig. 2F). Moreover, daumone effectively inhibited the age-associated upregulation of these fibrosis markers.

### Improved glucose intolerance and hepatic insulin sensitivity in daumone-treated aged mice

**Figure 3:**
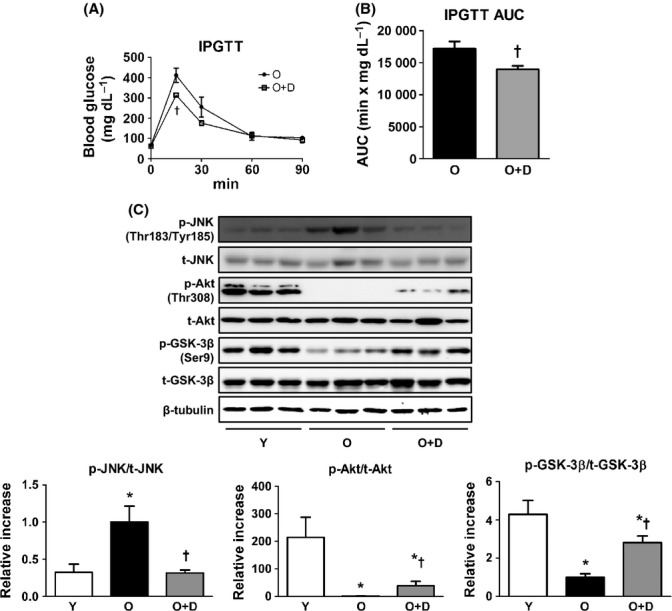
Effects of daumone on insulin sensitivity in aged mice. (A) Intraperitoneal glucose tolerance test (IPGTT) was performed at day 140 after daumone administration. (B) The area under the curve (AUC) for IPGTT was calculated. (C) Immunoblots were conducted and quantitated. Data are presented as the mean±SE of 7-17 mice/group. *P < 0.05 vs. Y, †*P* < 0.05 vs. O. JNK, c-Jun N-terminal kinase; GSK, glycogen synthase kinase; Y, young mice; O, old mice; O+D, old mice treated with daumone.

In aged mice, the plasma insulin levels are elevated, and insulin resistance is accelerated (Fan *et al*., 2011). To assess whether daumone could improve insulin insensitivity in old mice, intraperitoneal glucose tolerance test (IPGTT) was performed. At 4 months after daumone administration, the area under the curve (AUC) following an IPGTT was significantly decreased in daumone-treated aged mice as compared to untreated aged mice (Fig. 3A,B).

Next, to assess how daumone affects insulin signaling in the liver, the expression of proteins associated with insulin signaling was measured. c-Jun N-terminal kinase (JNK) phosphorylation was significantly increased in the livers of old mice compared with young mice, and JNK phosphorylation was decreased following daumone administration (Fig. 3C). In addition, the protein levels of the phosphorylated forms of Akt and glycogen synthase kinase-3β (GSK-3β) were significantly decreased in old mice compared with young mice (Fig. 3C), and the expression of these proteins was rescued by daumone. These results suggest that daumone improved systemic glucose intolerance and hepatic insulin sensitivity in aged mice.

### Ameliorated lipid accumulation in daumone-treated aged mice

**Figure 4:**
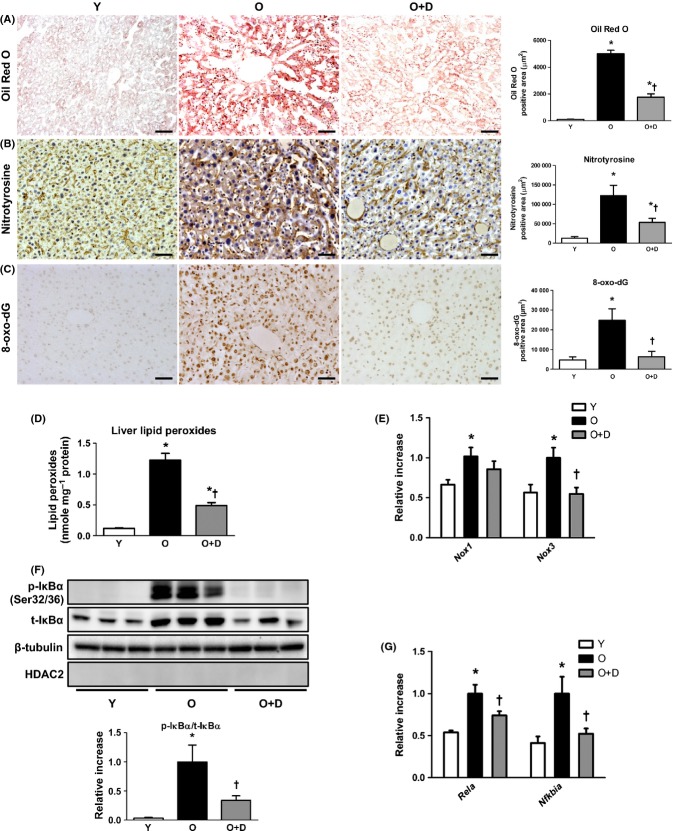
Effects of daumone on hepatic lipid accumulation and oxidative stress in aged mice. (A) Lipid droplets were stained with Oil Red O; blue, hematoxylin. Oxidative stress was determined by staining with (B) nitrotyrosine (brown; blue, hematoxylin) and (C) 8-hydroxy-2′-deoxyguanosine (8-oxo-dG) (brown). The positive area was quantitated using Image-Pro Plus and presented as the mean±SE of 4 mice/group. (D) Lipid peroxides were measured. (E, G) mRNA levels were determined by real-time qRT–PCR. (F) Immunoblots of the liver cytosolic fraction are presented and quantitated. Data in (D–G) are presented as the mean±SE of 7-17 mice/group. *P < 0.05 vs. Y, †*P* < 0.05 vs. O. IκB, inhibitor of nuclear factor-κB; Y, young mice; O, old mice; O+D, old mice treated with daumone. Magnification, 200×; scale bar, 50 μm.

To confirm the effect of daumone on lipid accumulation in the livers of old mice, Oil Red O staining was performed. Histological analysis revealed that the amount of lipid droplets in hepatocytes was significantly increased in old mice compared with young mice (Fig. 4A), and the extent of lipid droplets was effectively diminished by daumone. The mRNA levels of several genes associated with lipid metabolism were then measured in the liver, including *Ppara* and *Acadm* (regulators of peroxisomal β-oxidation), *Acox1* (a *Ppara* target gene), and *Pparg* (a regulator of lipogenesis). The levels of these genes were significantly decreased, whereas the levels of *Ppargc1a* (a mitochondrial biogenesis marker) and *Srebf2* (a regulator of transcription of the low-density lipoprotein receptor and fatty acids) were significantly increased in old mice compared with young mice (Fig. S1). The decreased hepatic mRNA levels detected for *Ppara*, *Acadm*, and *Acox1* were increased by daumone, whereas no significant differences were observed in the mRNA levels of *Cpt1a*, *Hmgcr*, *Srebpf1*, or *Fasn* in any of the experimental groups.

### Attenuated oxidative stress in daumone-treated aged mice

Oxidative stress is implicated in the pathogenesis of inflammation, fibrosis, and insulin resistance (Morgan & Liu, 2011). Accordingly, nitrotyrosine, 8-hydroxy-2′-deoxyguanosine (8-oxo-dG), and LPO, markers of oxidative stress, were greatly increased in the livers of old mice compared with young mice (Fig. 4B–D), which were attenuated by daumone. NADPH oxidase (NOX) enzymes, the catalytic subunit of NADPH oxidase, generate superoxide anions. The liver mRNA levels of *Nox1* and *Nox3* were significantly increased in old mice compared with young mice (Fig. 4E), and the mRNA levels of *Nox3* were significantly reduced by daumone.

To investigate whether daumone affected the antioxidant defense systems in the liver, the mRNA levels of *Sod1*, *Sod2*, *Cat*, *Gpx1*, *Nfe2 l2*, *Prx3*, and *Prx5* were measured (Fig. S2). The liver mRNA levels of *Sod1*, *Sod2*, *Cat*, and *Gpx1* were significantly reduced in old mice compared with young mice, and daumone significantly increased the mRNA levels of *Sod1* and *Cat*. No significant differences were observed in the expression of *Nfe2 l2*, *Prx3*, and *Prx5* in any of the experimental groups.

### Decreased inhibitor of nuclear factor-κB α (IκBα) phosphorylation in daumone-treated aged mice

Nuclear factor-κB (NF-κB), a redox-sensitive and oxidant-activated transcription factor, is a protein complex that controls the transcription of inflammation-related genes (Chung *et al*., 2002). Aged organs, including the liver, demonstrate decreased levels of IκBα in the cytosol but increased levels of NF-κB in the nucleus (Chung *et al*., 2002). In our experiments, we found that IκBα phosphorylation was increased in the cytosolic fraction of livers from aged mice compared with young mice (Fig. 4F), and phosphorylated IκBα was effectively decreased following daumone administration, suggesting that daumone may inhibit NF-κB activation in the aged liver. Consistently, the mRNA levels of *Rela* (p65-NF-κB) and *Nfkbia* (IκBα), a target of NF-κB (Pahl, 1999), were significantly increased in old mice compared with young mice, and daumone significantly reduced these mRNA levels (Fig. 4G).

### Attenuated LPS-induced liver injury by daumone in young mice

**Figure 5:**
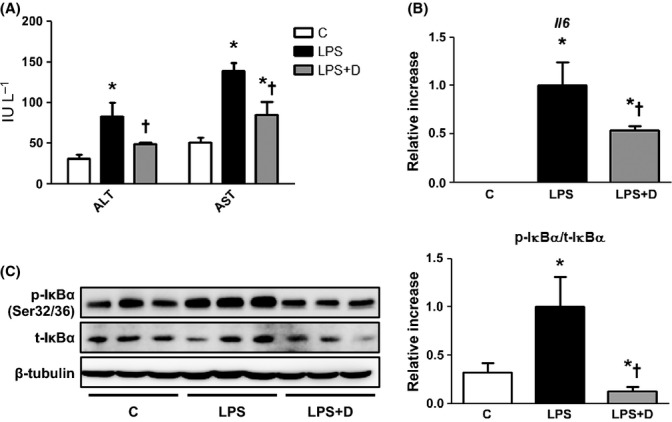
Effect of daumone on lipopolysaccharide (LPS)-induced liver injury in young mice. (A) Plasma alanine transaminase (ALT) and aspartate transaminase (AST) were measured. (B) The liver mRNA levels of *Il6* were determined by real-time qRT–PCR. (C) Immunoblots of IκBα in the liver cytosolic fraction are presented and quantitated. Data are presented as the mean±SE of 5 mice/group. *P < 0.05 vs. C, †*P* < 0.05 vs. LPS. IκBα, inhibitor of nuclear factor-κB α; C, control mice; LPS, mice injected with LPS (10 mg kg^−1^); LPS+D, LPS-injected mice treated with daumone (1 mg kg^−1^).

The effect of daumone on LPS-induced liver injury in young mice was determined to verify its anti-inflammatory effect. The levels of plasma ALT and AST (Fig. 5A) and hepatic *Il6* mRNA (Fig. 5B) were increased in LPS-treated mice, which was effectively inhibited by daumone pretreatment. Daumone also inhibited LPS-induced IκBα phosphorylation in the livers of mice (Fig. 5C).

## Discussion

The present study reveals daumone as a potential novel therapeutic agent for delaying aging and age-related liver disease. We found that daumone improved survival in mice and attenuated hepatic aging, possibly through its anti-inflammatory properties. Moreover, Kaplan–Meier analysis showed that daumone treatment reduced the risk of death in aged mice compared with untreated control mice. Although daumone has been shown to induce the dauer stage and increase the lifespan in *C. elegans*, this is the first study to demonstrate that daumone increases survival in mammals. In addition, it was previously reported that resveratrol administered to 12-month-old mice reduced the risk of death from a high-calorie diet (Baur *et al*., 2006) and increased the mean lifespan and the maximum lifespan (Pearson *et al*., 2008). SRT1720 fed to 6-month-old mice increased mean lifespan in mice fed a standard diet or a high-fat diet (Mitchell *et al*., 2014). Rapamycin is also a well-known drug, which increases the lifespan of mice (Harrison *et al*., 2009). Metformin fed to 12-month-old mice extended the mean lifespan (Martin-Montalvo *et al*., 2013). Additional studies with mice from diverse genetic backgrounds are required to compare the longevity effects of daumone, resveratrol, SRT1720, rapamycin, and metformin under the same experimental conditions. Although an absolute comparison is difficult, owing to diverse experimental conditions, it should be acknowledged that daumone is an orally active compound; in fact, daumone reduced the risk of death by 48% in normal diet-fed mice, even when administered to mice as late as 24 months of age.

Next, we focused on the hepatoprotective effects of daumone in aged mice. As expected, hepatic SA-β-gal activity, an index of cellular senescence, was increased in old mice but was decreased following daumone administration, suggesting that daumone may attenuate liver aging. Moreover, inflammation is closely related to aging (Freund *et al*., 2010), and daumone treatment effectively inhibited hepatic inflammation, including macrophage infiltration and the expression of proinflammatory cytokines. In mice, liver hypertrophy progresses with age, whereas in humans, liver mass decreases with age (Farahmand *et al*., 2013). Our results indicated that daumone inhibited hepatic inflammation and reduced age-induced liver hypertrophy. Daumone also effectively halted hepatic fibrosis and ECM accumulation. Consistent with the notion that hepatitis is a major cause of liver cirrhosis (Poynard *et al*., 2001), the present data demonstrate that at doses capable of inhibiting hepatic inflammation, daumone effectively inhibits fibrosis. Hepatic macrophage infiltration (estimated by F4/80 staining) and fibrosis (estimated by TGF-β1 and PAI-1) were further increased in 29-month-old mice compared with 25-month-old mice, which were effectively inhibited by daumone treatment (Fig. S3), suggesting that daumone may delay age-associated inflammation and fibrosis.

Inflammation also plays an important role in insulin resistance (Cai *et al*., 2005), and salicylates, anti-inflammatory agents, were shown to improve insulin sensitivity in obese mice (Yuan *et al*., 2001). Consistently, daumone improved age-associated insulin resistance at doses that inhibited inflammation. In the IPGTT, daumone improved systemic glucose intolerance in aged mice. Akt is activated by the insulin receptor through PI3K, and aging is known to induce insulin resistance and decrease Akt phosphorylation in the liver of 24- to 28-month-old rats (Shay & Hagen, 2009) and 20-month-old mice (Haga *et al*., 2010). Consistent with these previous reports, hepatic Akt phosphorylation and downstream GSK-3β phosphorylation were decreased in aged mice under the current experimental condition. Moreover, the levels of these proteins were increased following daumone administration, suggesting that daumone improved insulin resistance in the liver.

Given that fatty liver disease progresses to steatohepatitis (Reddy & Rao, 2006), we investigated whether daumone would affect lipid accumulation, mitochondrial biogenesis, and fatty acid metabolism. Lipids are accumulated in the aged mouse liver (Gregg *et al*., 2012), and the SIRT1 activator with anti-aging effect (Minor *et al*., 2011; Mitchell *et al*., 2014) increases fatty acid oxidation-related genes in the livers of high-fat-diet-fed mice (Feige *et al*., 2008). In agreement with these studies, the present study showed that increased lipid accumulation in old mouse livers was effectively reduced by daumone, and the fatty acid β-oxidation markers (*PPARa*, *Acadm*, and *Acox1* mRNA) that are generally decreased in livers from old mice were effectively increased following daumone treatment. However, daumone did not affect cholesterol synthesis markers such as *Pparg* and *Hmgcr*. Although the precise mechanism by which daumone affects lipid metabolism remains to be clarified, the daumone-induced upregulation of fatty acid β-oxidation could lead to decreased lipid accumulation.

Reactive oxygen species are increased in the livers of aged experimental animals, and this increase is a major factor leading to hepatic inflammation, in part through activation of the NF-κB pathway (Chung *et al*., 2002). In addition, the expression of antioxidant enzymes, such as catalase, is decreased in the aged liver (Mura *et al*., 1996). Consistent with previous studies, the plasma levels of LPO and hepatic levels of nitrotyrosine, 8-oxo-dG, LPO, and NOX3 mRNA were increased in aged livers compared with livers from young mice in the present study, whereas the levels of antioxidant enzymes were decreased in aged livers. Thus, daumone reduced oxidative stress and increased the levels of antioxidant enzymes.

Finally, we examined the NF-κB activation state and the underlying mechanism for the anti-inflammatory effects of daumone. Daumone inhibited IκBα phosphorylation and upregulation of *Rela* and *Nfkbia* mRNA expression in the aged liver, suggesting that daumone may inhibit NF-κB activation. In previous studies, nuclear NF-κB binding and NF-κB-p65 protein expression levels in both the nuclear and cytosolic fractions were increased in aged rat livers (Helenius *et al*., 2001), and transgenic mice carrying a constitutively active liver-specific form of IκB kinase 2 (IKK2) exhibited increased IκBα phosphorylation, leading to increased NF-κB activation and inflammation (Sunami *et al*., 2012). Our data that daumone protected LPS-induced liver injury including hepatic IκBα phosphorylation in young mice further support the notion that daumone has anti-aging effect through its anti-inflammatory properties.

It is necessary to determine the receptor or target of daumone in mammals. In this connection, it has been demonstrated that the dauer pheromone binds to a specific cell surface G-protein-coupled receptor (GPCR), resulting in activation of the G-protein α-subunits GPA-2 and GPA-3 (homolog of mammalian G-protein G(o) α-2 and α-1, respectively) in amphid neurons (Zwaal *et al*., 1997). Putative GPCR for daumone has not yet been verified in mammals. *In vitro* kinase assay suggests that daumone may not directly inhibit kinases involved in inflammatory pathways including GSK3α, GSK3β, IKKα, IKKβ, JAK1, JNK1α1, and JNK2α2 (unpublished data). Our observation that daumone decreased plasma triglycerides and ameliorated decreased hepatic mRNA levels of *Acox1* (a PPARα target gene) as well as *PPARa* in old mice suggests that daumone may act as a PPARα agonist. Future studies regarding the role of PPARα in daumone’s anti-aging effects are needed.

In summary, the present study demonstrated that daumone improves survival in aged mice and provides anti-aging effects, including decreased liver hypertrophy, senescence, fibrosis, and lipid accumulation. Daumone in aged mice also improved hepatic insulin signaling and systemic insulin sensitivity and attenuated hepatic inflammation and macrophage infiltration, possibly through the suppression of NF-κB signaling. These results suggest that daumone may represent a novel therapeutic molecule for aging-induced pathology and age-related diseases. Therefore, uncovering the detailed mechanisms responsible for these daumone-mediated anti-inflammatory effects may promote effective interventions targeting chronic inflammation, aging, and age-related diseases.

## Experimental procedures

All chemicals were obtained from Sigma-Aldrich Co. (St. Louis, MO, USA) and tissue culture plates from NUNC (Roskilde, Denmark) and Becton Dickinson Labware (Lincoln Park, NJ, USA), unless otherwise stated.

### Daumone

Daumone was obtained according to the previous report (Jeong *et al*., 2005). Briefly, stereospecific total synthesis of the pheromone was successfully performed in 10 steps by coupling the key intermediate, 2,4-di-O-benzyl-3,6-dideoxy-L-arabino-hexopyranose, synthesized starting from L-rhamnose and (2R)-7-octene-2-ol. The purity was confirmed by NMR as more than 95.6% pure.

### Experimental animals and daumone treatment

All animal experiments were conducted according to the Institutional Animal Care and Use Committee (IACUC) of Ewha Womans University (2010-27-1). Our previous studies confirmed that orally administered daumone in drinking water was detected in rats (Noh *et al*., 2011) and mice (Noh *et al*., 2012). In the latter study, plasma daumone concentration was 74 ng mL^−1^ at 2 h after 5 weeks of repeated oral administration of daumone 2 mg kg^−1^ day^−1^ to mice. It was confirmed that the daumone was absorbed and distributed into mice plasma.

Six-month-old C57BL/6J male mice were purchased from Charles River Laboratory (Shizuoka, Japan), housed in a room maintained at 22 ± 2 °C, exposed to a 12-h dark/12-h light cycle, and fed a standard chow diet (PMI® Nutrition International, LLC Certified Rodent LABDIET® 5053, Purina Mills, Richmond, IN, USA) and tap water *ad libitum*. Mice were maintained until the age of 24 months (old mice), and their body weights were monitored every month. Old mice were randomly divided into two groups, which received either daumone in drinking water (2 mg kg^−1^ day^−1^) or the same volume of drinking water by oral gavage for 5 months. Each group of old mice initially contained 32 animals. Calorie intake was little changed (176–203 kcal kg^−1^ body weight day^−1^), and no difference in body weight occurred in response to daumone treatment. Nine-week-old control mice were purchased and killed 1 week later. Mice in fed state were anesthetized with avertin (intraperitoneal injection, i.p., 0.3 g kg^−1^), and blood was collected. Livers were collected and immediately stored at −70 °C.

### LPS-induced liver injury

Six-week-old C57BL/6J male mice were purchased from Charles River Laboratory (Shizuoka, Japan). Mice were randomly divided into three groups (control, LPS, and LPS+D). LPS+D mice were treated with daumone (1 mg kg^−1^; i.p.) for 10 days; control and LPS mice received saline. After the last injection of daumone or saline, LPS and LPS+D mice were injected with LPS (phenol extracted from *E*. coli serotype O111:B4, Sigma-Aldrich, Co., 10 mg kg^−1^, i.p.). At 7 h after LPS administration, mice were killed. The protocol was approved by IACUC of Ewha Womans University (ELAGC-09-1025).

### Intraperitoneal glucose tolerance test (IPGTT)

Intraperitoneal glucose tolerance test (IPGTT) was performed by injecting glucose (2 g kg^−1^) after 18 h of food deprivation. Blood samples were taken from the tail vein to measure blood glucose levels before and at 15, 30, 60, and 90 min after glucose administration, using a ONETOUCH Ultra glucose meter (LIFESCAN, Burnaby, Canada).

### Measurements of blood parameters and enzyme-linked immunosorbent assay (ELISA)

The blood HbA1c level was measured using the DCA2000 HbA1c reagent kit (SIEMENS Healthcare Diagnostics, Inc., Tarrytown, NY, USA). The blood was centrifuged at 900 ***g*** for 15 min at 4 °C, and the supernatant was collected. The levels of plasma ALT, cholesterols, triglycerides, and free fatty acids were measured using an EnzyChrom™ colorimetric assay kit (BioAssay Systems, Hayward, CA, USA). The levels of plasma insulin were measured using ELISA kits (Millipore Corporation, Billerica, MA, USA) according to the manufacturer’s protocol. The plasma and the liver levels of malondialdehyde, as detected by a reaction with thiobarbituric acid, were used as an index of the level of LPO. Plasma and the livers were mixed with 0.8% (w/v) thiobarbituric acid in acetic acid and incubated for 1 h in boiling water. After cooling, the absorbance of malondialdehyde was measured at an excitation of 555 nm and an emission at 515 nm. The levels of malondialdehyde were determined based on a standard curve generated with 1,1,3,3-tetraethoxypropane as the standard. TGF-β1 protein expression in the livers was assessed with commercially available ELISA kits (R&D Systems, Minneapolis, MN, USA) according to the manufacturer’s recommendations. The intra-assay coefficient of variation (CV) was 2.8%, and the inter-assay CV was 7.5%.

### Morphometric and immunohistochemical analysis

Livers were fixed in 4% formalin, dehydrated, and embedded in paraffin for sectioning. The liver tissue sections were deparaffinized and stained with Masson’s modified trichrome, according to the manufacturer’s protocol. Immunohistochemistry was performed with immunoperoxidase procedures and a commercially available kit (Dakocytomation, Glostrup, Denmark). Briefly, the sections were deparaffinized, and the endogenous peroxidase activity was quenched with Dako peroxidase solution. Then, the sections were incubated with Dako serum-free blocking solution prior to incubation with anti-nitrotyrosine (1:200), anti-F4/80 (1:200), and anti-8-oxo-dG (1:200) antibodies overnight at 4 °C. The sections incubated with the anti-nitrotyrosine antibody were subsequently incubated with a horseradish peroxidase-conjugated anti-mouse IgG secondary antibody (1:200, Santa Cruz Biotechnology, Santa Cruz, CA, USA). The sections incubated with the anti-F4/80 antibody and anti-8-oxo-dG were subsequently incubated with the rat and mouse ABC staining kit (Santa Cruz Biotechnology and Dakocytomation), respectively. After washing with PBS, the sections were exposed to 3,3′-diaminobenzidine. Digital images were captured on a Zeiss microscope equipped with an Axio Cam HRC digital camera (Carl Zeiss, Thornwood, NY, USA), and the positive staining area in 10 fields (100× magnification, 200× for Masson’s trichrome) per animal was analyzed with Image-Pro Plus 4.5.1 (Media Cybernetics, Silver Springs, MD, USA). The antibodies used are listed in Table S2 (Supporting information).

### Detection of SA-β-gal activity

SA-β-gal activity was detected using a senescence detection kit (BioVision, Mountain View, CA, USA) according to the manufacturer’s protocol (Dimri *et al*., 1995). Briefly, frozen liver tissue sections fixed in 4% formalin were warmed to room temperature and incubated overnight at 37 °C with fresh SA-β-gal staining solution (470 μl of staining solution, 5 μl of staining supplement, 25 μl of 20 mg mL^−1^ X-gal in DMF). After washing with PBS, images of the sections were captured with a Zeiss microscope and analyzed using the open-source image analysis program imagej v. 1.34s (Rasband, WS, ImageJ, US National Institutes of Health, Bethesda, MD, USA).

### Real-time quantitative reverse transcription PCR (qRT–PCR)

A standard real-time qRT–PCR was performed. Total RNA was extracted from tissues using TRIzol (Invitrogen, Carlsbad, CA, USA). The mRNA levels were assessed by real-time qRT–PCR using the SYBR Green PCR Master Mix kit (Applied Biosystems, Foster City, CA, USA) with an ABI 7300 Real-time qRT-PCR thermal cycler (Applied Biosystems), as described previously (Song *et al*., 2013). The relative levels of the test genes and the internal control 18S ribosomal RNA (rRNA) were determined using a standard curve produced with the applied biosystems software. The primer sequences are shown in Table S1 (Supporting information).

### Western blot analysis

The protein levels in liver tissue homogenates were measured using a standard Western blotting protocol (Song *et al*., 2013), as described previously. Cytosolic fraction was isolated (Hattori *et al*., 1990) when needed. The protein concentrations of the samples were determined with the Bradford assay (Bio-Rad Laboratories, Hercules, CA, USA). Equal amounts of protein from each sample were mixed with loading buffer and separated on SDS–PAGE gels by electrophoresis. The proteins were then transferred onto a polyvinylidene fluoride membrane (GE Healthcare BioSciences, Piscataway, NJ, USA), and the membranes were incubated overnight at 4 °C with a 1:2000 dilution of various antibodies. Next, the membranes were incubated with peroxidase-conjugated secondary antibodies, and then the signals were visualized with an enhanced chemiluminescence system detection reagent (GE Healthcare BioSciences). Positive immunoreactive bands were quantitated with a densitometer (LAS-3000, FUJIFILM, Tokyo, Japan) and normalized to the levels of total protein and β-tubulin. The antibodies used are listed in Table S2 (Supporting information).

### Oil Red O staining

Hepatic neutral lipid was detected by a standard Oil Red O staining. Frozen liver tissue sections fixed in 4% formalin were warmed to room temperature. After washing with PBS, the sections were incubated in 100% propylene glycol for 10 min and then stained with 0.7% Oil Red O in propylene glycol. The Oil Red O dye was then washed off with 85% propylene glycol. Images of the sections were captured with a Zeiss microscope and analyzed using Image-Pro Plus.

### Statistical analysis

All results are expressed as the mean ± standard error (SE). Survival curves were analyzed using the Kaplan–Meier method. A Cox regression analysis was used for calculating the risk of death. Analysis of variance (anova) was used to assess differences between multiple groups. If the *F* statistic was significant, the mean values of each group were compared with the Fisher’s least significant difference method. A *P* value <0.05 was considered statistically significant.

## Supporting Information

**Fig. S1** Daumone treatment altered lipid metabolism. **Fig. S2** Daumone treatment increased the expression of antioxidant enzymes. **Fig. S3** Inflammation and fibrosis were increased in the livers of aged mice. **Table S1** Primer sequences. **Table S2** List of antibodies.
