# Supplementary material for: Daumone fed late in life improves survival and reduces hepatic inflammation and fibrosis in mice
Source: Aging Cell. 2014 May 6;13(4):709–18. doi: 10.1111/acel.12224 (PMC4326938; doi:10.1111/acel.12224)
Supplement: Supplementary file 1 — Fig. S1 Daumone treatment altered lipid metabolism. Fig. S2 Daumone treatment increased the expression of antioxidant enzymes. Fig. S3 Inflammation and fibrosis were increased in the livers of aged mice. Table S1 Primer sequences. Table S2 List of antibodies. [file acel0013-0709-sd1.docx]

**Park JH et al.: Daumone fed late in life improves survival and reduces hepatic inflammation and fibrosis in mice**

**Additional experimental procedures in supporting information**

Supporting tables: 2

Supporting figures: 3

**Additional experimental procedures in supporting information**

**Experimental animals**

**Twenty five-month-old mice:** All animal experiments were conducted according to the Institutional Animal Care and Use Committee of Ewha Womans University (2010-27-1). Six-month-old C57BL/6J male mice were purchased from Charles River Laboratory (Shizuoka, Japan). Mice were maintained until the age of 25 months (25M) and sacrificed.

**Table S1 Primer sequences**

| **Genes** | **Forward (5’-3’)** | **Reverse (5’-3’)** | **Size (bp)** |
| --- | --- | --- | --- |
| *Rn18s* | CGA AAG CAT TTG CCA AGA AT | AGT CGG CAT CGT TTA TGG TC | 267 |
| *Acadm* | CAA CAC TCG AAA GCG GCT CA | ACT TGC GGG CAG TTG CTT G | 97 |
| *Acox1* | AGA GCC CCA GAT TTT GGA TT | TCC GCA TGT AGG TCT CCT TT | 113 |
| *Acta2* | GTC CCA GAC ATC AGG GAG TAA | TCG GAT ACT TCA GCG TCA GGA | 102 |
| *Cat* | CAC ACC TA ACG CAG GCC GG | CTG CGC TCC GGA GTG GGA GA | 156 |
| *Ccl2* | CTT CTG GGC CTG CTG TTC A | CCA GCC TAC TCA TTG GGA TCA | 127 |
| *Col1a1* | CGG ATA GCA GAT TGA GAA CAT CCG | CGG CTG AGT ACG GAA CAC ACA | 202 |
| *Col4a1* | ATT CCT TCG TGA TGC ACA CC | GTG GGC TTC TTG AAC ATC TC | 196 |
| *Cpt1a* | ACC ACT GGC CGC ATG TCA AG | AGC GAG TAG CGC ATA GTC AT | 101 |
| *Emr1* | CTG TAA CCG GAT GGC AAA CT | ATG GCC AAG GCA AGA CAT AC | 123 |
| *Fasn* | CCT GGA TAG CAT TCC GAA CCT | GCA CAT CTC GAA GGC TAC ACA | 121 |
| *Gpx1* | GCC AAC ACC CAG TGA CGA CCC | GCG GCA CAC CGG AGA CCA AAT | 73 |
| *Hmgcr* | GTG CCC TAA ATT TGA AGA GG | GTA GGT TCT GGA ACT GGA AG | 104 |
| *Icam1* | AGG TGG TTC TTC TGA GCG GC | AAA CAG GAA CTT TCC CGC CA | 137 |
| *Il6* | AGT TGC CTT CTT GGG ACT GA | TCC ACG ATT TCC CAG AGA AC | 159 |
| *Nfe2l2* | CTC TCT GAA CTC CTG GAC GG | GGG TCT CCG TAA ATG GAA G | 182 |
| *Nfkbia* | CTG CAG GCC ACC AAC TAC AA | CAG CAC CCA AAG TCA CCA AGT | 97 |
| *Nos2* | GGC AGC CTG TGA GAC CTT TG | CAT TGG AAG TGA AGC GTT TCG | 71 |
| *Nox1* | GGG TGG GT TGT CCG GGG TCA | TGC CCC GCA AAA TGA GGA TGC C | 125 |
| *Nox3* | ACC GTG GAG GAG GCA ATT AGA CAA | CAG GTT GAA GAA ATG CGC CAC GAT | 106 |
| *Ppara* | TCA GGG TAC CAC TAC GGA GTT CA | CCG AAT TCG CCG AAA GA | 66 |
| *Pparg* | GGT GAA ACT CTG GGA GAT TC | CAA CCA TTG GGT CAG CTC TT | 268 |
| *Ppargc1a* | TCG ATG TGT CGC CTT CTT GC | ACG AGA GCG CAT CCT TTG G | 104 |
| *Prdx3* | GCG GCT GCG GGA AGG TTG CT | TGC TGG GTG ACA GCA GGG GT | 200 |
| *Prdx5* | CCA AGG GAG G AGG TGG TG | GCC TTC TGC CTG GG GGC TC | 85 |
| *Ptgs2* | GTG GAA AAA CCT CGT CCA GA | GCT CGG CTT CCA GTA TTG AG | 120 |
| *Rela* | CTG ATG TGC ATC GGC AAG TG | AGC TGC ATG GAG ACT CGA AC | 91 |

**Table S1 Primer sequences (*****continued*)**

| **Genes** | **Forward (5’-3’)** | **Reverse (5’-3’)** | **Size (bp)** |
| --- | --- | --- | --- |
| *Serpine1* | AGG GCT TCA TGC CCC ACT TCT TCA | AGT AGA GGG CAT TCA CCA GCA CCA | 192 |
| *Sod1* | GTG TGG GTG CTG AAG GGC GA | GAC GTG GAA CC ATG CTG GCC | 159 |
| *Sod2* | CCG AGG AGA AGT ACC ACG AG | GCT TGA TAG CCT CCA GCA AC | 174 |
| *Srebpf1* | TGT GGA GCT CAA AGA CCT G | TGT GCT GCA AGA AGC GGA TG | 98 |
| *Srebf2* | CAA GTC TGG CGT TCT GAG GAA | ATG TTC TCC TGG CGC AGC T | 81 |
| *Tgfb1* | CTT TAG GAA GGA CCT GGG TT | CAG GAC CGC ACA ATC ATG TT | 258 |
| *Tnf* | CGT CAG CCG ATT TGC TAT CT | CGG ACT CCG CAA AGT CTA AG | 206 |

**Table S2 List of antibodies**

| **Antidodies** | **Phpsphorylation site** | **Size (kDa)** | **Company** | Cat No. |
| --- | --- | --- | --- | --- |
| p-JNK | Thr183/Tyr185 | 46, 54 | Cell Signaling Technology | 9251 |
| t-JNK |  | 46, 54 | Cell Signaling Technology | 9252 |
| p-Akt | Thr308 | 60 | Cell Signaling Technology | 9275 |
| t-Akt |  | 60 | Cell Signaling Technology | 9272 |
| F4/80 |  | - | Santa Cruz Biotechnology | SC-71088 |
| p-GSK-3β | Ser9 | 46 | Cell Signaling Technology | 9323 |
| t-GSK-3β |  | 46 | Cell Signaling Technology | 9315 |
| HDAC2 |  | 59 | Santa Cruz Biotechnology | SC-7899 |
| p-IκBα | Ser32/36 | 40 | Cell Signaling Technology | 9246 |
| t-IκBα |  | 40 | Santa Cruz Biotechnology | SC-371 |
| ICAM-1 |  | 85-110 | Santa Cruz Biotechnology | SC-1511 |
| Nitrotyrosine |  | - | Santa Cruz Biotechnology | SC-32757 |
| 8-oxo-dG |  | - | Trevigen | 4354-MC-050 |
| α-SMA |  | 42 | Sigma-Aldrich Co. | A5228 |
| β-tubulin |  | 55 | Santa Cruz Biotechnology | SC-9104 |

p-JNK, phosphorylated c-Jun N-terminal kinase; p-GSK-3β, phosphorylated glycogen synthase kinase-3β; p-IκBα, phosphorylated inhibitor of nuclear factor-κB α; ICAM-1, Intercellular adhesion molecule-1; HDAC2, histone deacetylase 2; 8-oxo-dG, 8-hydroxy-2'-deoxyguanosine; α-SMA, α-smooth muscle actin; Cell Signaling Technology, Danvers, MA, USA; Santa Cruz Biotechnology, Santa Cruz, CA, USA; Sigma-Aldrich Co., St. Louis, MO, USA; Trevigen, Gaithersburg, MD, USA


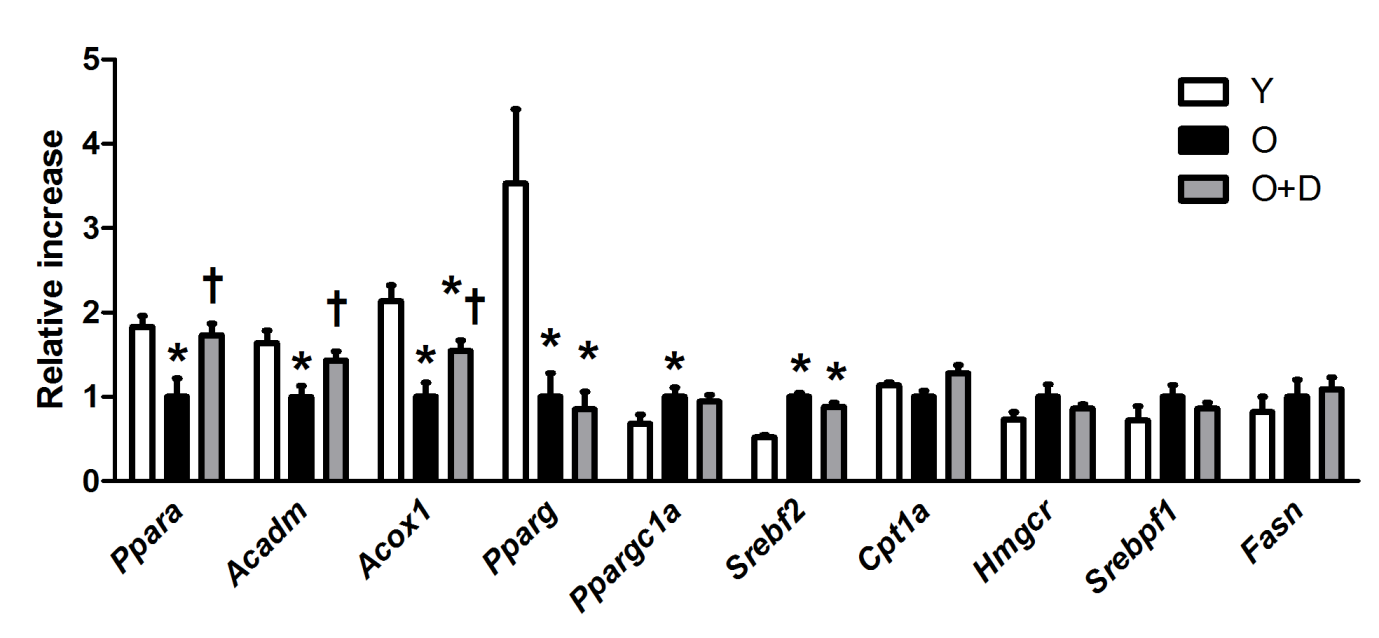


**Fig. S1 Daumone treatment altered lipid metabolism.**

The liver mRNA levels of *Ppara*, *Acadm*, *Acox1*, *Pparg*, *Ppargc1a*, *Srebf2*, *Cpt1a, Hmgcr*, *Srebpf1*, and *Fasn* were determined by real-time qRT-PCR. Data are presented as the mean±SE of 7-17 mice/group. *p<0.05 vs. Y, †p<0.05 vs. O. *p<0.05 vs. Y †p<0.05 vs. O. Y, young mice; O, old mice; O+D, old mice treated with daumone (2 mg/kg).


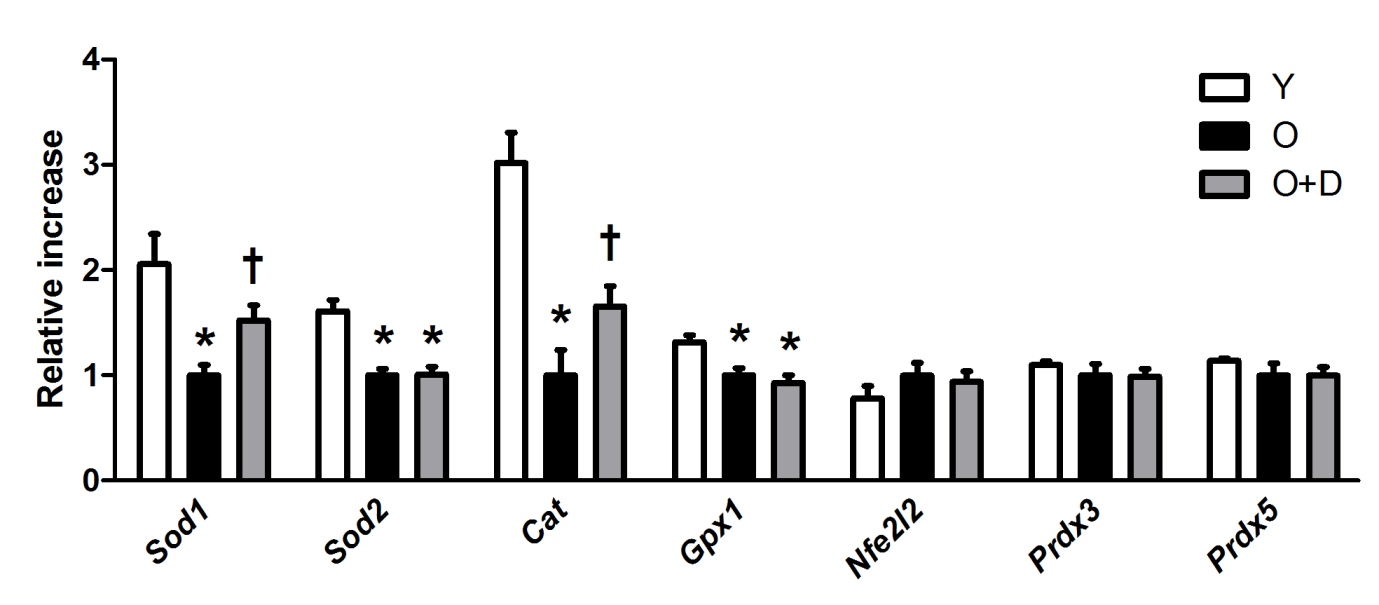


**Fig. S2 Daumone treatment increased the expression of antioxidant enzymes.**

The liver mRNA levels of *Sod1*, *Sod2*, *Cat*, *Gpx1*, *Nfe2l2*, *Prx3*, and *Prx5* were determined by real-time qRT-PCR. Data are presented as the mean±SE of 7-17 mice/group. *p<0.05 vs. Y, †p<0.05 vs. O. Y, young mice; O, old mice; O+D, old mice treated with daumone (2 mg/kg).

**
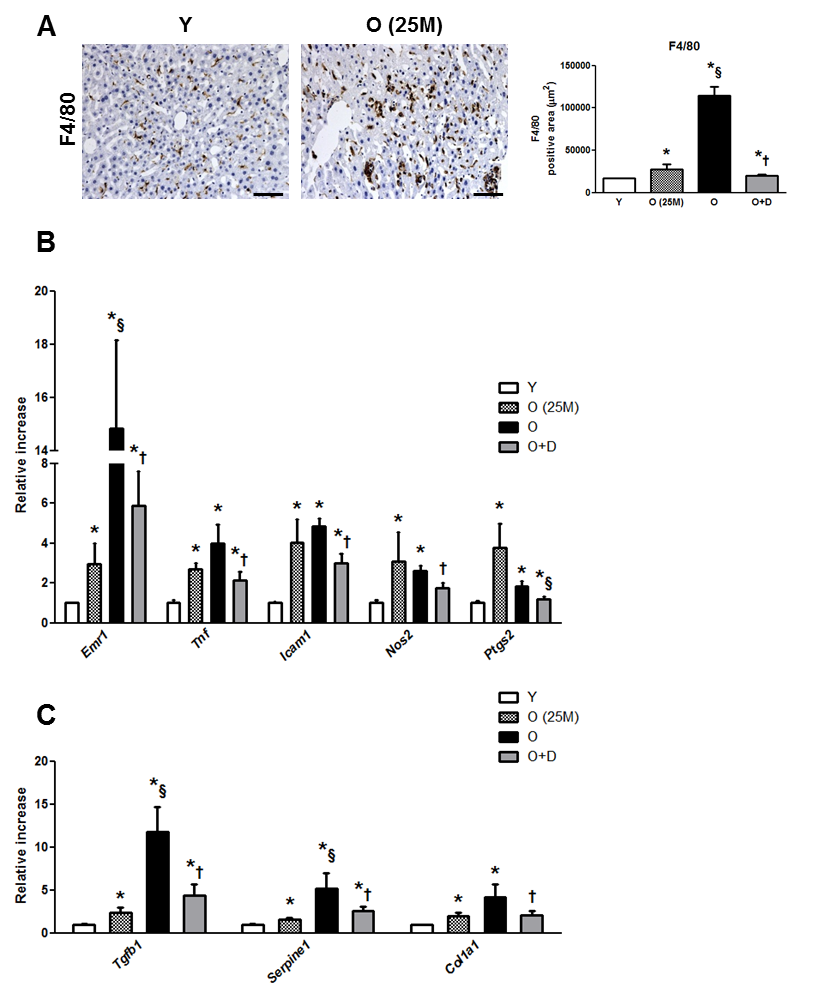
**

**Fig. S3 Inflammation and fibrosis were increased in the livers of aged mice.**

(A) Macrophage infiltration was detected by F4/80 immunohistochemical staining. Brown, F4/80; blue, hematoxylin. The positive area was quantified using Image-Pro Plus and presented as the mean±SE of 3 mice/group. The liver mRNA levels of (B) *Emr1*, *Tnf*, *Icam1*, *Nos2*, and *Ptgs2* and (C) *Tgfb1*, *Serpine1*, and *Col1a1* were determined by real-time qRT-PCR and presented as the mean±SE of 7-17 mice/group. *p<0.05 vs. Y, §p<0.05 vs. O (25M), †p<0.05 vs. O. Y, young mice; O (25M), 25-month-old mice; O, old mice; O+D, old mice treated with daumone (2 mg/kg).
